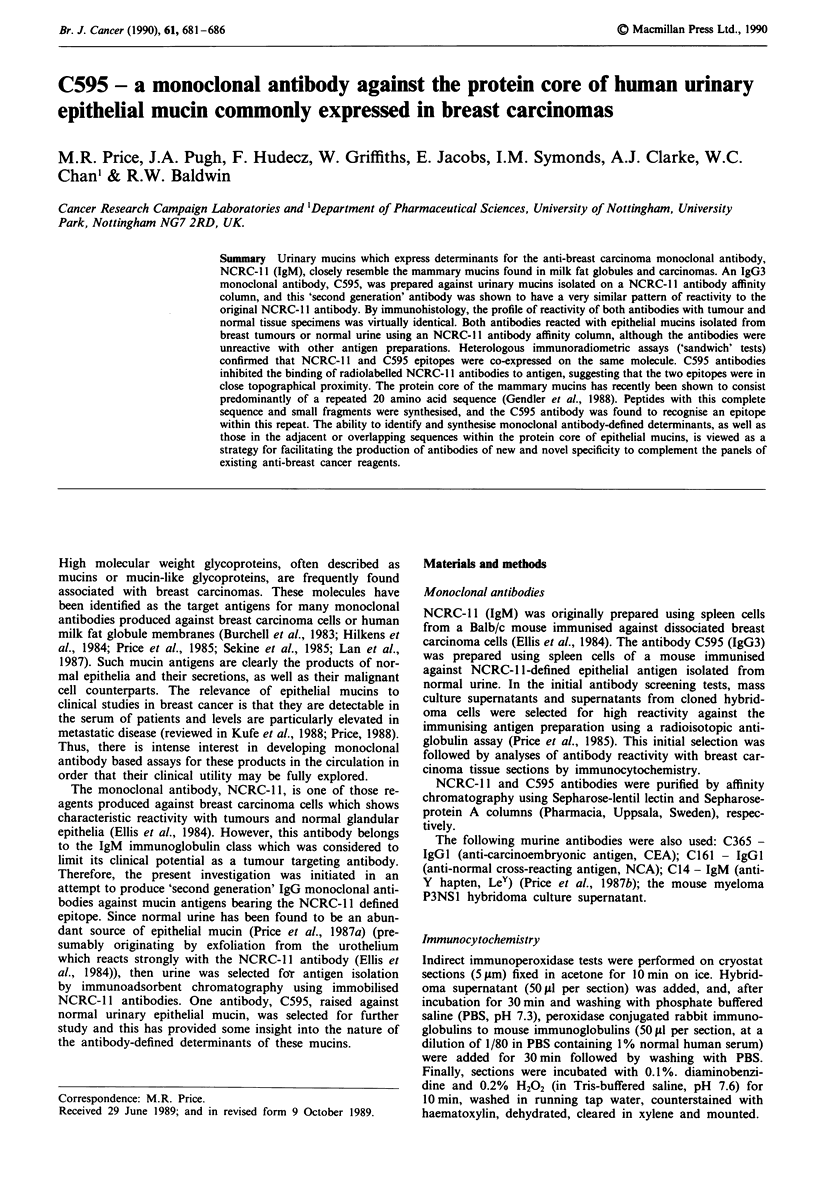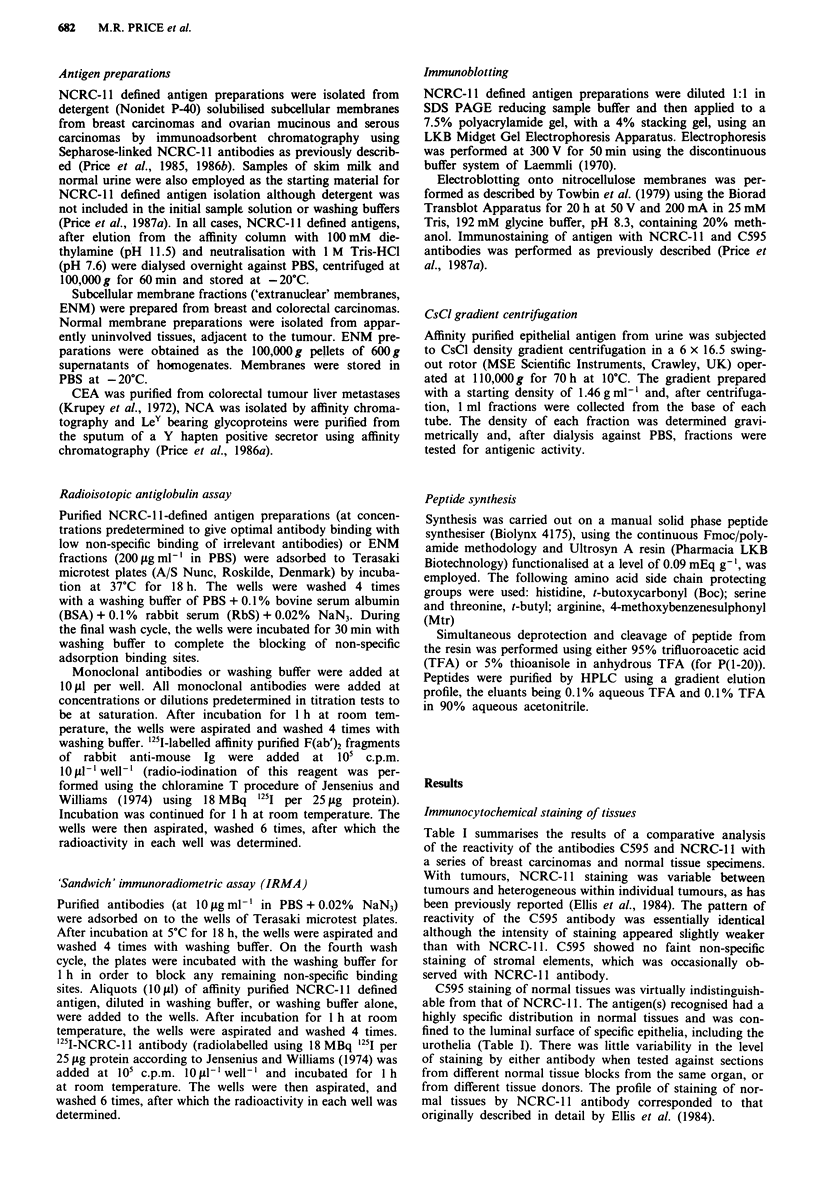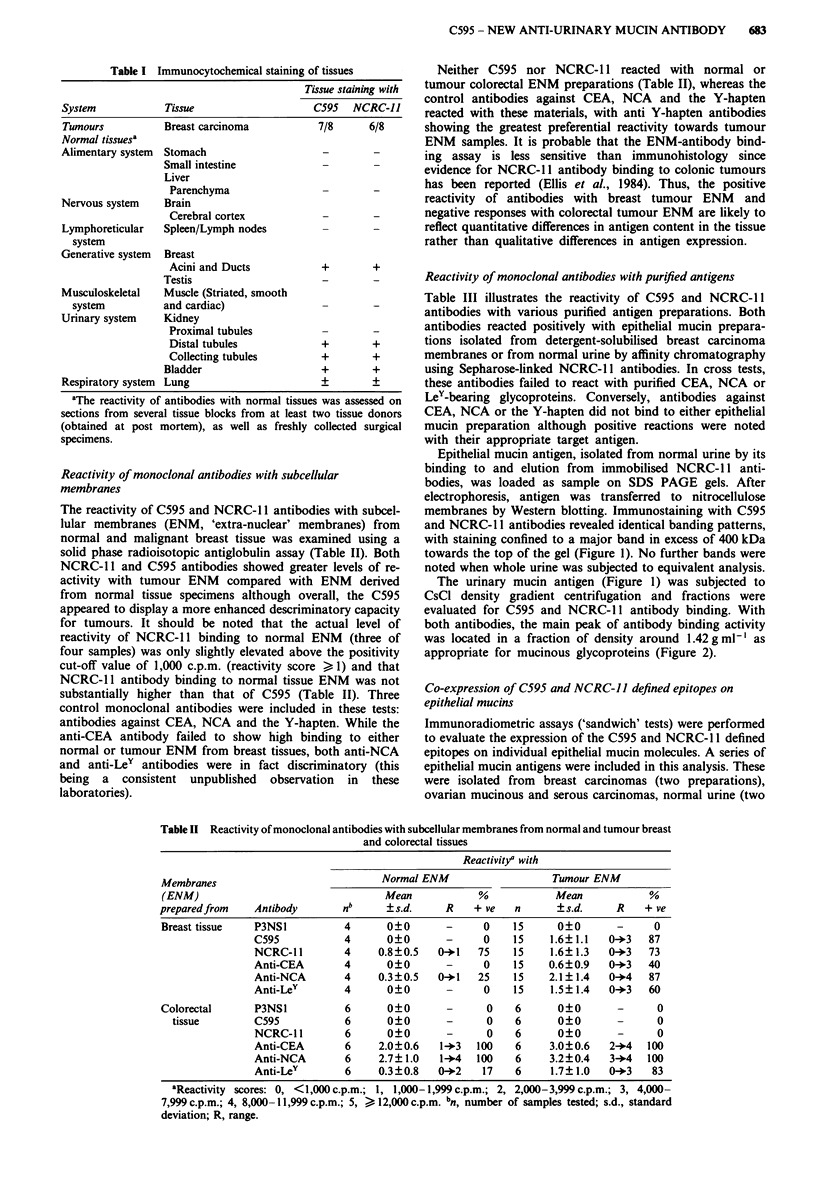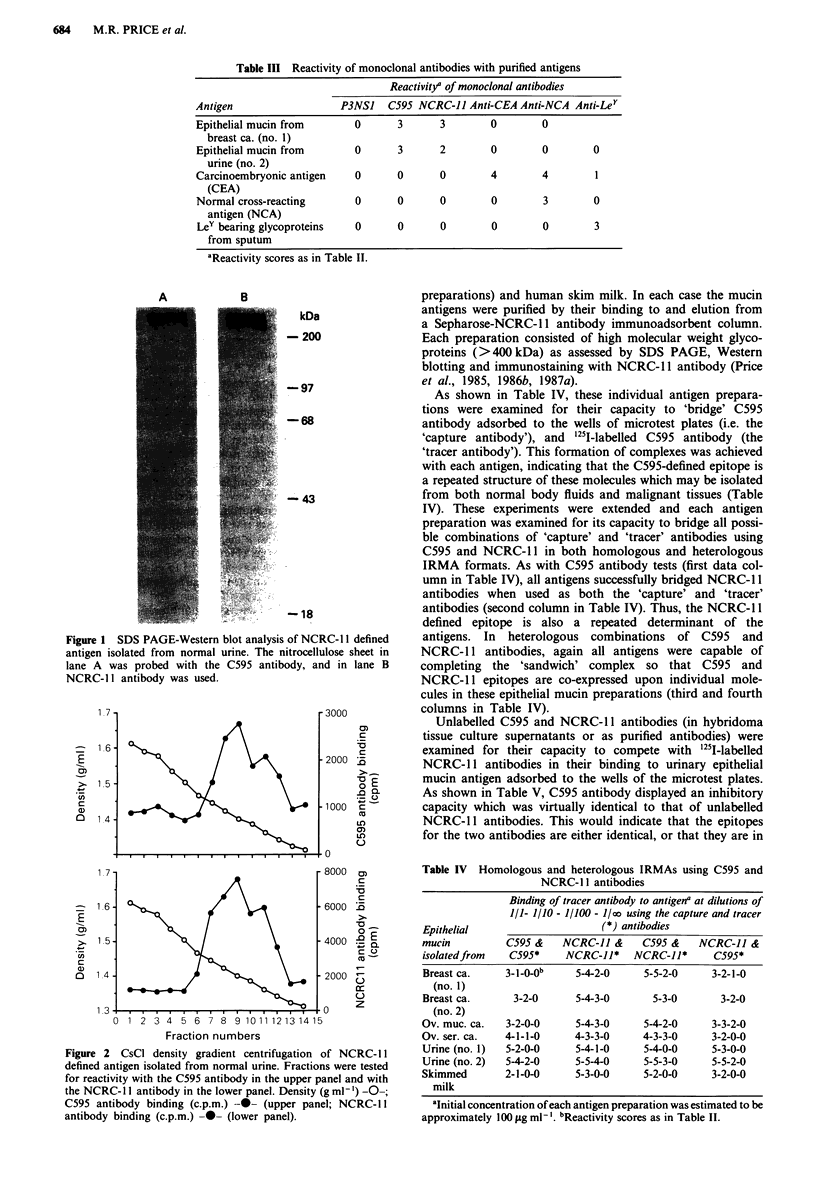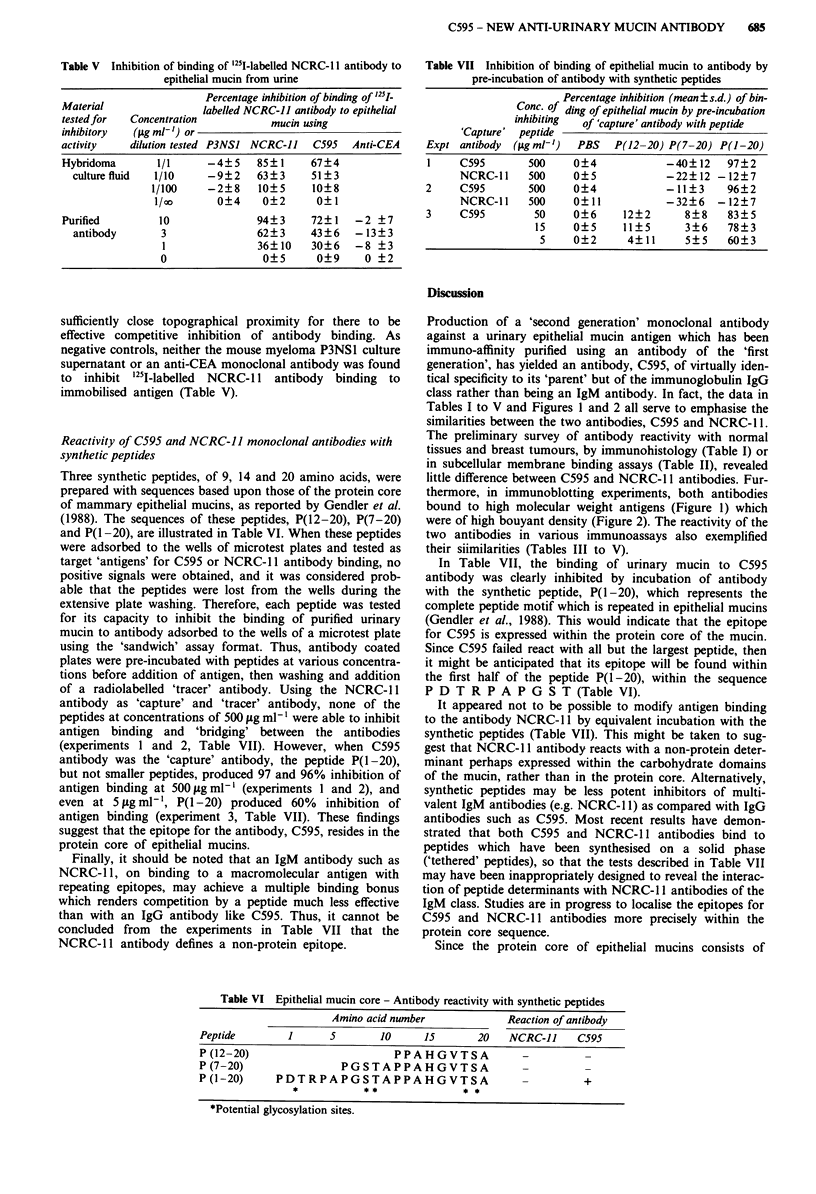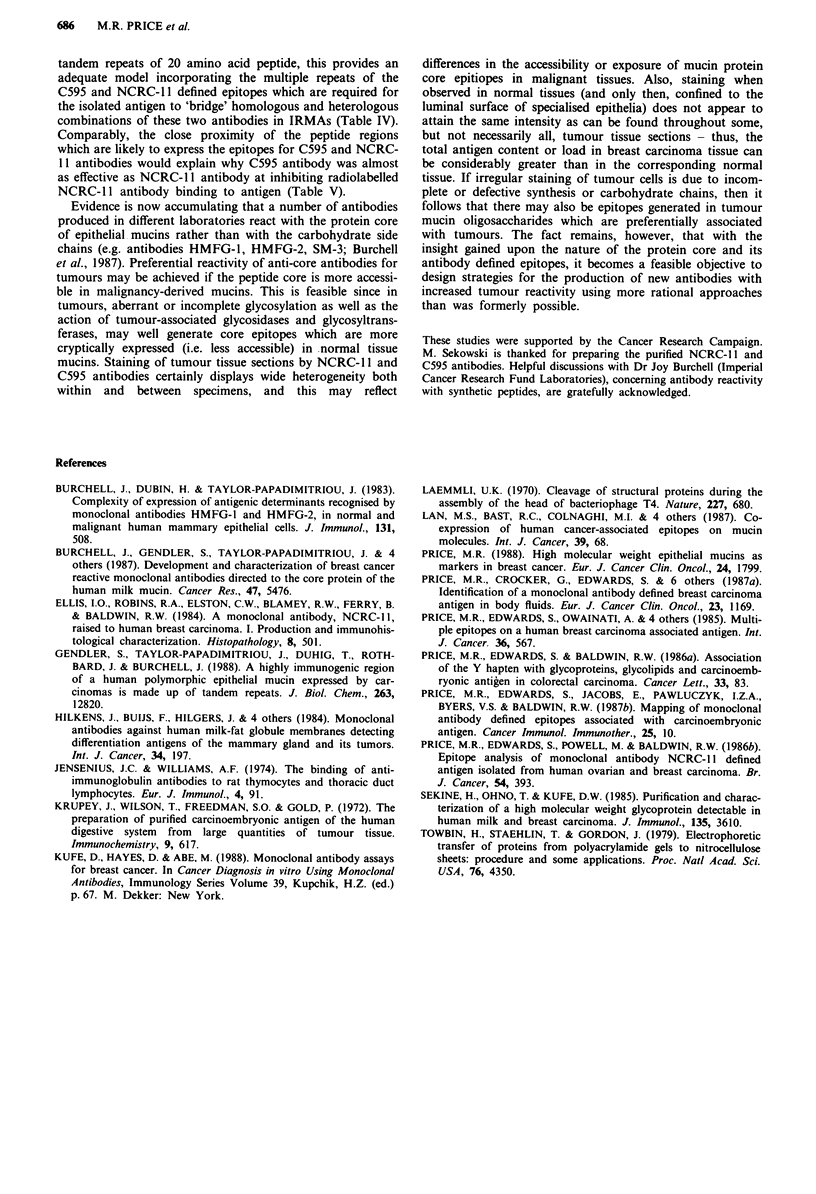# C595--a monoclonal antibody against the protein core of human urinary epithelial mucin commonly expressed in breast carcinomas.

**DOI:** 10.1038/bjc.1990.154

**Published:** 1990-05

**Authors:** M. R. Price, J. A. Pugh, F. Hudecz, W. Griffiths, E. Jacobs, I. M. Symonds, A. J. Clarke, W. C. Chan, R. W. Baldwin

**Affiliations:** Cancer Research Campaign Laboratories, University of Nottingham, University Park, UK.

## Abstract

**Images:**


Br. J. Cancer (1990), 61, 681-686 © Macmillan Press Ltd., 1990

### C595 – a monoclonal antibody against the protein core of human urinary epithelial mucin commonly expressed in breast carcinomas

M.R. Price, J.A. Pugh, F. Hudecz, W. Griffiths, E. Jacobs, I.M. Symonds, A.J. Clarke, W.C. Chan[1] & R.W. Baldwin

*Cancer Research Campaign Laboratories and [1]Department of Pharmaceutical Sciences, University of Nottingham, University Park, Nottingham NG7 2RD, UK.*

**Summary** Urinary mucins which express determinants for the anti-breast carcinoma monoclonal antibody, NCRC-11 (IgM), closely resemble the mammary mucins found in milk fat globules and carcinomas. An IgG3 monoclonal antibody, C595, was prepared against urinary mucins isolated on a NCRC-11 antibody affinity column, and this 'second generation' antibody was shown to have a very similar pattern of reactivity to the original NCRC-11 antibody. By immunohistology, the profile of reactivity of both antibodies with tumour and normal tissue specimens was virtually identical. Both antibodies reacted with epithelial mucins isolated from breast tumours or normal urine using an NCRC-11 antibody affinity column, although the antibodies were unreactive with other antigen preparations. Heterologous immunoradiometric assays ('sandwich' tests) confirmed that NCRC-11 and C595 epitopes were co-expressed on the same molecule. C595 antibodies inhibited the binding of radiolabelled NCRC-11 antibodies to antigen, suggesting that the two epitopes were in close topographical proximity. The protein core of the mammary mucins has recently been shown to consist predominantly of a repeated 20 amino acid sequence (Gendler *et al.*, 1988). Peptides with this complete sequence and small fragments were synthesised, and the C595 antibody was found to recognise an epitope within this repeat. The ability to identify and synthesise monoclonal antibody-defined determinants, as well as those in the adjacent or overlapping sequences within the protein core of epithelial mucins, is viewed as a strategy for facilitating the production of antibodies of new and novel specificity to complement the panels of existing anti-breast cancer reagents.

High molecular weight glycoproteins, often described as mucins or mucin-like glycoproteins, are frequently found associated with breast carcinomas. These molecules have been identified as the target antigens for many monoclonal antibodies produced against breast carcinoma cells or human milk fat globule membranes (Burchell *et al.*, 1983; Hilkens *et al.*, 1984; Price *et al.*, 1985; Sekine *et al.*, 1985; Lan *et al.*, 1987). Such mucin antigens are clearly the products of normal epithelia and their secretions, as well as their malignant cell counterparts. The relevance of epithelial mucins to clinical studies in breast cancer is that they are detectable in the serum of patients and levels are particularly elevated in metastatic disease (reviewed in Kufe *et al.*, 1988; Price, 1988). Thus, there is intense interest in developing monoclonal antibody based assays for these products in the circulation in order that their clinical utility may be fully explored.

The monoclonal antibody, NCRC-11, is one of those reagents produced against breast carcinoma cells which shows characteristic reactivity with tumours and normal glandular epithelia (Ellis *et al.*, 1984). However, this antibody belongs to the IgM immunoglobulin class which was considered to limit its clinical potential as a tumour targeting antibody. Therefore, the present investigation was initiated in an attempt to produce 'second generation' IgG monoclonal antibodies against mucin antigens bearing the NCRC-11 defined epitope. Since normal urine has been found to be an abundant source of epithelial mucin (Price *et al.*, 1987*a*) (presumably originating by exfoliation from the urothelium which reacts strongly with the NCRC-11 antibody (Ellis *et al.*, 1984)), then urine was selected for antigen isolation by immunoadsorbent chromatography using immobilised NCRC-11 antibodies. One antibody, C595, raised against normal urinary epithelial mucin, was selected for further study and this has provided some insight into the nature of the antibody-defined determinants of these mucins.

Correspondence: M.R. Price.
Received 29 June 1989; and in revised form 9 October 1989.

### Materials and methods

### Monoclonal antibodies

NCRC-11 (IgM) was originally prepared using spleen cells from a Balb/c mouse immunised against dissociated breast carcinoma cells (Ellis *et al.*, 1984). The antibody C595 (IgG3) was prepared using spleen cells of a mouse immunised against NCRC-11-defined epithelial antigen isolated from normal urine. In the initial antibody screening tests, mass culture supernatants and supernatants from cloned hybridoma cells were selected for high reactivity against the immunising antigen preparation using a radioisotopic antiglobulin assay (Price *et al.*, 1985). This initial selection was followed by analyses of antibody reactivity with breast carcinoma tissue sections by immunocytochemistry.

NCRC-11 and C595 antibodies were purified by affinity chromatography using Sepharose-lentil lectin and Sepharose-protein A columns (Pharmacia, Uppsala, Sweden), respectively.

The following murine antibodies were also used: C365 – IgG1 (anti-carcinoembryonic antigen, CEA); C161 – IgG1 (anti-normal cross-reacting antigen, NCA); C14 – IgM (anti-Y hapten, $Le^Y$) (Price *et al.*, 1987*b*); the mouse myeloma P3NS1 hybridoma culture supernatant.

### Immunocytochemistry

Indirect immunoperoxidase tests were performed on cryostat sections (5 μm) fixed in acetone for 10 min on ice. Hybridoma supernatant (50 μl per section) was added, and, after incubation for 30 min and washing with phosphate buffered saline (PBS, pH 7.3), peroxidase conjugated rabbit immunoglobulins to mouse immunoglobulins (50 μl per section, at a dilution of 1/80 in PBS containing 1% normal human serum) were added for 30 min followed by washing with PBS. Finally, sections were incubated with 0.1%. diaminobenzidine and 0.2% $H_2O_2$ (in Tris-buffered saline, pH 7.6) for 10 min, washed in running tap water, counterstained with haematoxylin, dehydrated, cleared in xylene and mounted.

*Antigen preparations*

NCRC-11 defined antigen preparations were isolated from detergent (Nonidet P-40) solubilised subcellular membranes from breast carcinomas and ovarian mucinous and serous carcinomas by immunoadsorbent chromatography using Sepharose-linked NCRC-11 antibodies as previously described (Price *et al.*, 1985, 1986*b*). Samples of skim milk and normal urine were also employed as the starting material for NCRC-11 defined antigen isolation although detergent was not included in the initial sample solution or washing buffers (Price *et al.*, 1987*a*). In all cases, NCRC-11 defined antigens, after elution from the affinity column with 100 mM diethylamine (pH 11.5) and neutralisation with 1 M Tris-HCl (pH 7.6) were dialysed overnight against PBS, centrifuged at 100,000 *g* for 60 min and stored at −20°C.

Subcellular membrane fractions ('extranuclear' membranes, ENM) were prepared from breast and colorectal carcinomas. Normal membrane preparations were isolated from apparently uninvolved tissues, adjacent to the tumour. ENM preparations were obtained as the 100,000 *g* pellets of 600 *g* supernatants of homogenates. Membranes were stored in PBS at −20°C.

CEA was purified from colorectal tumour liver metastases (Krupey *et al.*, 1972), NCA was isolated by affinity chromatography and $Le^Y$ bearing glycoproteins were purified from the sputum of a Y hapten positive secretor using affinity chromatography (Price *et al.*, 1986*a*).

*Radioisotopic antiglobulin assay*

Purified NCRC-11-defined antigen preparations (at concentrations predetermined to give optimal antibody binding with low non-specific binding of irrelevant antibodies) or ENM fractions (200 $\mu g\ ml^{-1}$ in PBS) were adsorbed to Terasaki microtest plates (A/S Nunc, Roskilde, Denmark) by incubation at 37°C for 18 h. The wells were washed 4 times with a washing buffer of PBS + 0.1% bovine serum albumin (BSA) + 0.1% rabbit serum (RbS) + 0.02% $NaN_3$. During the final wash cycle, the wells were incubated for 30 min with washing buffer to complete the blocking of non-specific adsorption binding sites.

Monoclonal antibodies or washing buffer were added at 10 $\mu l$ per well. All monoclonal antibodies were added at concentrations or dilutions predetermined in titration tests to be at saturation. After incubation for 1 h at room temperature, the wells were aspirated and washed 4 times with washing buffer. $^{125}I$-labelled affinity purified $F(ab')_2$ fragments of rabbit anti-mouse Ig were added at $10^5$ c.p.m. 10 $\mu l^{-1}$ $well^{-1}$ (radio-iodination of this reagent was performed using the chloramine T procedure of Jensenius and Williams (1974) using 18 MBq $^{125}I$ per 25 $\mu g$ protein). Incubation was continued for 1 h at room temperature. The wells were then aspirated, washed 6 times, after which the radioactivity in each well was determined.

*'Sandwich' immunoradiometric assay (IRMA)*

Purified antibodies (at 10 $\mu g\ ml^{-1}$ in PBS + 0.02% $NaN_3$) were adsorbed on to the wells of Terasaki microtest plates. After incubation at 5°C for 18 h, the wells were aspirated and washed 4 times with washing buffer. On the fourth wash cycle, the plates were incubated with the washing buffer for 1 h in order to block any remaining non-specific binding sites. Aliquots (10 $\mu l$) of affinity purified NCRC-11 defined antigen, diluted in washing buffer, or washing buffer alone, were added to the wells. After incubation for 1 h at room temperature, the wells were aspirated and washed 4 times. $^{125}I$-NCRC-11 antibody (radiolabelled using 18 MBq $^{125}I$ per 25 $\mu g$ protein according to Jensenius and Williams (1974) was added at $10^5$ c.p.m. 10 $\mu l^{-1}$ $well^{-1}$ and incubated for 1 h at room temperature. The wells were then aspirated, and washed 6 times, after which the radioactivity in each well was determined.

*Immunoblotting*

NCRC-11 defined antigen preparations were diluted 1:1 in SDS PAGE reducing sample buffer and then applied to a 7.5% polyacrylamide gel, with a 4% stacking gel, using an LKB Midget Gel Electrophoresis Apparatus. Electrophoresis was performed at 300 V for 50 min using the discontinuous buffer system of Laemmli (1970).

Electroblotting onto nitrocellulose membranes was performed as described by Towbin *et al.* (1979) using the Biorad Transblot Apparatus for 20 h at 50 V and 200 mA in 25 mM Tris, 192 mM glycine buffer, pH 8.3, containing 20% methanol. Immunostaining of antigen with NCRC-11 and C595 antibodies was performed as previously described (Price *et al.*, 1987*a*).

*CsCl gradient centrifugation*

Affinity purified epithelial antigen from urine was subjected to CsCl density gradient centrifugation in a 6 × 16.5 swing-out rotor (MSE Scientific Instruments, Crawley, UK) operated at 110,000 *g* for 70 h at 10°C. The gradient prepared with a starting density of 1.46 $g\ ml^{-1}$ and, after centrifugation, 1 ml fractions were collected from the base of each tube. The density of each fraction was determined gravimetrically and, after dialysis against PBS, fractions were tested for antigenic activity.

*Peptide synthesis*

Synthesis was carried out on a manual solid phase peptide synthesiser (Biolynx 4175), using the continuous Fmoc/polyamide methodology and Ultrosyn A resin (Pharmacia LKB Biotechnology) functionalised at a level of 0.09 mEq $g^{-1}$, was employed. The following amino acid side chain protecting groups were used: histidine, *t*-butoxycarbonyl (Boc); serine and threonine, *t*-butyl; arginine, 4-methoxybenzenesulphonyl (Mtr)

Simultaneous deprotection and cleavage of peptide from the resin was performed using either 95% trifluoroacetic acid (TFA) or 5% thioanisole in anhydrous TFA (for P(1-20)). Peptides were purified by HPLC using a gradient elution profile, the eluants being 0.1% aqueous TFA and 0.1% TFA in 90% aqueous acetonitrile.

### Results

*Immunocytochemical staining of tissues*

Table I summarises the results of a comparative analysis of the reactivity of the antibodies C595 and NCRC-11 with a series of breast carcinomas and normal tissue specimens. With tumours, NCRC-11 staining was variable between tumours and heterogeneous within individual tumours, as has been previously reported (Ellis *et al.*, 1984). The pattern of reactivity of the C595 antibody was essentially identical although the intensity of staining appeared slightly weaker than with NCRC-11. C595 showed no faint non-specific staining of stromal elements, which was occasionally observed with NCRC-11 antibody.

C595 staining of normal tissues was virtually indistinguishable from that of NCRC-11. The antigen(s) recognised had a highly specific distribution in normal tissues and was confined to the luminal surface of specific epithelia, including the urothelia (Table I). There was little variability in the level of staining by either antibody when tested against sections from different normal tissue blocks from the same organ, or from different tissue donors. The profile of staining of normal tissues by NCRC-11 antibody corresponded to that originally described in detail by Ellis *et al.* (1984).

**Table I** Immunocytochemical staining of tissues

| System | Tissue | Tissue staining with C595 | NCRC-11 |
|---|---|---|---|
| *Tumours* | Breast carcinoma | 7/8 | 6/8 |
| *Normal tissues*[a] | | | |
| Alimentary system | Stomach | – | – |
| | Small intestine | – | – |
| | Liver | | |
| | Parenchyma | – | – |
| Nervous system | Brain | | |
| | Cerebral cortex | – | – |
| Lymphoreticular system | Spleen/Lymph nodes | – | – |
| Generative system | Breast | | |
| | Acini and Ducts | + | + |
| | Testis | – | – |
| Musculoskeletal system | Muscle (Striated, smooth and cardiac) | – | – |
| Urinary system | Kidney | | |
| | Proximal tubules | – | – |
| | Distal tubules | + | + |
| | Collecting tubules | + | + |
| | Bladder | + | + |
| Respiratory system | Lung | ± | ± |

[a]The reactivity of antibodies with normal tissues was assessed on sections from several tissue blocks from at least two tissue donors (obtained at post mortem), as well as freshly collected surgical specimens.

### *Reactivity of monoclonal antibodies with subcellular membranes*

The reactivity of C595 and NCRC-11 antibodies with subcellular membranes (ENM, 'extra-nuclear' membranes) from normal and malignant breast tissue was examined using a solid phase radioisotopic antiglobulin assay (Table II). Both NCRC-11 and C595 antibodies showed greater levels of reactivity with tumour ENM compared with ENM derived from normal tissue specimens although overall, the C595 appeared to display a more enhanced descriminatory capacity for tumours. It should be noted that the actual level of reactivity of NCRC-11 binding to normal ENM (three of four samples) was only slightly elevated above the positivity cut-off value of 1,000 c.p.m. (reactivity score $\geqslant 1$) and that NCRC-11 antibody binding to normal tissue ENM was not substantially higher than that of C595 (Table II). Three control monoclonal antibodies were included in these tests: antibodies against CEA, NCA and the Y-hapten. While the anti-CEA antibody failed to show high binding to either normal or tumour ENM from breast tissues, both anti-NCA and anti-$Le^Y$ antibodies were in fact discriminatory (this being a consistent unpublished observation in these laboratories).

Neither C595 nor NCRC-11 reacted with normal or tumour colorectal ENM preparations (Table II), whereas the control antibodies against CEA, NCA and the Y-hapten reacted with these materials, with anti Y-hapten antibodies showing the greatest preferential reactivity towards tumour ENM samples. It is probable that the ENM-antibody binding assay is less sensitive than immunohistology since evidence for NCRC-11 antibody binding to colonic tumours has been reported (Ellis *et al.*, 1984). Thus, the positive reactivity of antibodies with breast tumour ENM and negative responses with colorectal tumour ENM are likely to reflect quantitative differences in antigen content in the tissue rather than qualitative differences in antigen expression.

### *Reactivity of monoclonal antibodies with purified antigens*

Table III illustrates the reactivity of C595 and NCRC-11 antibodies with various purified antigen preparations. Both antibodies reacted positively with epithelial mucin preparations isolated from detergent-solubilised breast carcinoma membranes or from normal urine by affinity chromatography using Sepharose-linked NCRC-11 antibodies. In cross tests, these antibodies failed to react with purified CEA, NCA or $Le^Y$-bearing glycoproteins. Conversely, antibodies against CEA, NCA or the Y-hapten did not bind to either epithelial mucin preparation although positive reactions were noted with their appropriate target antigen.

Epithelial mucin antigen, isolated from normal urine by its binding to and elution from immobilised NCRC-11 antibodies, was loaded as sample on SDS PAGE gels. After electrophoresis, antigen was transferred to nitrocellulose membranes by Western blotting. Immunostaining with C595 and NCRC-11 antibodies revealed identical banding patterns, with staining confined to a major band in excess of 400 kDa towards the top of the gel (Figure 1). No further bands were noted when whole urine was subjected to equivalent analysis.

The urinary mucin antigen (Figure 1) was subjected to CsCl density gradient centrifugation and fractions were evaluated for C595 and NCRC-11 antibody binding. With both antibodies, the main peak of antibody binding activity was located in a fraction of density around 1.42 g ml$^{-1}$ as appropriate for mucinous glycoproteins (Figure 2).

### *Co-expression of C595 and NCRC-11 defined epitopes on epithelial mucins*

Immunoradiometric assays ('sandwich' tests) were performed to evaluate the expression of the C595 and NCRC-11 defined epitopes on individual epithelial mucin molecules. A series of epithelial mucin antigens were included in this analysis. These were isolated from breast carcinomas (two preparations), ovarian mucinous and serous carcinomas, normal urine (two

**Table II** Reactivity of monoclonal antibodies with subcellular membranes from normal and tumour breast and colorectal tissues

| Membranes (ENM) prepared from | Antibody | Normal ENM $n^b$ | Normal ENM Mean ±s.d. | Normal ENM R | Normal ENM % + ve | Tumour ENM n | Tumour ENM Mean ±s.d. | Tumour ENM R | Tumour ENM % + ve |
|---|---|---|---|---|---|---|---|---|---|
| Breast tissue | P3NS1 | 4 | 0±0 | – | 0 | 15 | 0±0 | – | 0 |
| | C595 | 4 | 0±0 | – | 0 | 15 | 1.6±1.1 | 0→3 | 87 |
| | NCRC-11 | 4 | 0.8±0.5 | 0→1 | 75 | 15 | 1.6±1.3 | 0→3 | 73 |
| | Anti-CEA | 4 | 0±0 | – | 0 | 15 | 0.6±0.9 | 0→3 | 40 |
| | Anti-NCA | 4 | 0.3±0.5 | 0→1 | 25 | 15 | 2.1±1.4 | 0→4 | 87 |
| | Anti-$Le^Y$ | 4 | 0±0 | – | 0 | 15 | 1.5±1.4 | 0→3 | 60 |
| Colorectal tissue | P3NS1 | 6 | 0±0 | – | 0 | 6 | 0±0 | – | 0 |
| | C595 | 6 | 0±0 | – | 0 | 6 | 0±0 | – | 0 |
| | NCRC-11 | 6 | 0±0 | – | 0 | 6 | 0±0 | – | 0 |
| | Anti-CEA | 6 | 2.0±0.6 | 1→3 | 100 | 6 | 3.0±0.6 | 2→4 | 100 |
| | Anti-NCA | 6 | 2.7±1.0 | 1→4 | 100 | 6 | 3.2±0.4 | 3→4 | 100 |
| | Anti-$Le^Y$ | 6 | 0.3±0.8 | 0→2 | 17 | 6 | 1.7±1.0 | 0→3 | 83 |

[a]Reactivity scores: 0, <1,000 c.p.m.; 1, 1,000–1,999 c.p.m.; 2, 2,000–3,999 c.p.m.; 3, 4,000–7,999 c.p.m.; 4, 8,000–11,999 c.p.m.; 5, $\geqslant$12,000 c.p.m. [b]*n*, number of samples tested; s.d., standard deviation; R, range.

**Table III** Reactivity of monoclonal antibodies with purified antigens

| | *Reactivity[a] of monoclonal antibodies* | | | | | |
|---|---|---|---|---|---|---|
| *Antigen* | *P3NS1* | *C595* | *NCRC-11* | *Anti-CEA* | *Anti-NCA* | *Anti-Le$^Y$* |
| Epithelial mucin from breast ca. (no. 1) | 0 | 3 | 3 | 0 | 0 | |
| Epithelial mucin from urine (no. 2) | 0 | 3 | 2 | 0 | 0 | 0 |
| Carcinoembryonic antigen (CEA) | 0 | 0 | 0 | 4 | 4 | 1 |
| Normal cross-reacting antigen (NCA) | 0 | 0 | 0 | 0 | 3 | 0 |
| Le$^Y$ bearing glycoproteins from sputum | 0 | 0 | 0 | 0 | 0 | 3 |

[a]Reactivity scores as in Table II.

**Figure 1** SDS PAGE-Western blot analysis of NCRC-11 defined antigen isolated from normal urine. The nitrocellulose sheet in lane A was probed with the C595 antibody, and in lane B NCRC-11 antibody was used.

**Figure 2** CsCl density gradient centrifugation of NCRC-11 defined antigen isolated from normal urine. Fractions were tested for reactivity with the C595 antibody in the upper panel and with the NCRC-11 antibody in the lower panel. Density (g ml$^{-1}$) -O-; C595 antibody binding (c.p.m.) -●- (upper panel; NCRC-11 antibody binding (c.p.m.) -●- (lower panel).

preparations) and human skim milk. In each case the mucin antigens were purified by their binding to and elution from a Sepharose-NCRC-11 antibody immunoadsorbent column. Each preparation consisted of high molecular weight glycoproteins (>400 kDa) as assessed by SDS PAGE, Western blotting and immunostaining with NCRC-11 antibody (Price *et al*., 1985, 1986*b*, 1987*a*).

As shown in Table IV, these individual antigen preparations were examined for their capacity to 'bridge' C595 antibody adsorbed to the wells of microtest plates (i.e. the 'capture antibody'), and $^{125}$I-labelled C595 antibody (the 'tracer antibody'). This formation of complexes was achieved with each antigen, indicating that the C595-defined epitope is a repeated structure of these molecules which may be isolated from both normal body fluids and malignant tissues (Table IV). These experiments were extended and each antigen preparation was examined for its capacity to bridge all possible combinations of 'capture' and 'tracer' antibodies using C595 and NCRC-11 in both homologous and heterologous IRMA formats. As with C595 antibody tests (first data column in Table IV), all antigens successfully bridged NCRC-11 antibodies when used as both the 'capture' and 'tracer' antibodies (second column in Table IV). Thus, the NCRC-11 defined epitope is also a repeated determinant of the antigens. In heterologous combinations of C595 and NCRC-11 antibodies, again all antigens were capable of completing the 'sandwich' complex so that C595 and NCRC-11 epitopes are co-expressed upon individual molecules in these epithelial mucin preparations (third and fourth columns in Table IV).

Unlabelled C595 and NCRC-11 antibodies (in hybridoma tissue culture supernatants or as purified antibodies) were examined for their capacity to compete with $^{125}$I-labelled NCRC-11 antibodies in their binding to urinary epithelial mucin antigen adsorbed to the wells of the microtest plates. As shown in Table V, C595 antibody displayed an inhibitory capacity which was virtually identical to that of unlabelled NCRC-11 antibodies. This would indicate that the epitopes for the two antibodies are either identical, or that they are in

**Table IV** Homologous and heterologous IRMAs using C595 and NCRC-11 antibodies

| | *Binding of tracer antibody to antigen[a] at dilutions of 1/1- 1/10 - 1/100 - 1/∞ using the capture and tracer (*) antibodies* | | | |
|---|---|---|---|---|
| *Epithelial mucin isolated from* | *C595 & C595** | *NCRC-11 & NCRC-11** | *C595 & NCRC-11** | *NCRC-11 & C595** |
| Breast ca. (no. 1) | 3-1-0-0[b] | 5-4-2-0 | 5-5-2-0 | 3-2-1-0 |
| Breast ca. (no. 2) | 3-2-0 | 5-4-3-0 | 5-3-0 | 3-2-0 |
| Ov. muc. ca. | 3-2-0-0 | 5-4-3-0 | 5-4-2-0 | 3-3-2-0 |
| Ov. ser. ca. | 4-1-1-0 | 4-3-3-0 | 4-3-3-0 | 3-2-0-0 |
| Urine (no. 1) | 5-2-0-0 | 5-4-1-0 | 5-4-0-0 | 5-3-0-0 |
| Urine (no. 2) | 5-4-2-0 | 5-5-4-0 | 5-5-3-0 | 5-5-2-0 |
| Skimmed milk | 2-1-0-0 | 5-3-0-0 | 5-2-0-0 | 3-2-0-0 |

[a]Initial concentration of each antigen preparation was estimated to be approximately 100 μg ml$^{-1}$. [b]Reactivity scores as in Table II.

**Table V** Inhibition of binding of $^{125}$I-labelled NCRC-11 antibody to epithelial mucin from urine

| *Material tested for inhibitory activity* | *Concentration (µg ml$^{-1}$) or dilution tested* | *Percentage inhibition of binding of $^{125}$I-labelled NCRC-11 antibody to epithelial mucin using* P3NS1 | NCRC-11 | C595 | Anti-CEA |
|---|---|---|---|---|---|
| Hybridoma culture fluid | 1/1 | −4±5 | 85±1 | 67±4 | |
| | 1/10 | −9±2 | 63±3 | 51±3 | |
| | 1/100 | −2±8 | 10±5 | 10±8 | |
| | 1/∞ | 0±4 | 0±2 | 0±1 | |
| Purified antibody | 10 | | 94±3 | 72±1 | −2 ±7 |
| | 3 | | 62±3 | 43±6 | −13±3 |
| | 1 | | 36±10 | 30±6 | −8 ±3 |
| | 0 | | 0±5 | 0±9 | 0 ±2 |

sufficiently close topographical proximity for there to be effective competitive inhibition of antibody binding. As negative controls, neither the mouse myeloma P3NS1 culture supernatant or an anti-CEA monoclonal antibody was found to inhibit $^{125}$I-labelled NCRC-11 antibody binding to immobilised antigen (Table V).

*Reactivity of C595 and NCRC-11 monoclonal antibodies with synthetic peptides*

Three synthetic peptides, of 9, 14 and 20 amino acids, were prepared with sequences based upon those of the protein core of mammary epithelial mucins, as reported by Gendler *et al.* (1988). The sequences of these peptides, P(12–20), P(7–20) and P(1–20), are illustrated in Table VI. When these peptides were adsorbed to the wells of microtest plates and tested as target 'antigens' for C595 or NCRC-11 antibody binding, no positive signals were obtained, and it was considered probable that the peptides were lost from the wells during the extensive plate washing. Therefore, each peptide was tested for its capacity to inhibit the binding of purified urinary mucin to antibody adsorbed to the wells of a microtest plate using the 'sandwich' assay format. Thus, antibody coated plates were pre-incubated with peptides at various concentrations before addition of antigen, then washing and addition of a radiolabelled 'tracer' antibody. Using the NCRC-11 antibody as 'capture' and 'tracer' antibody, none of the peptides at concentrations of 500 µg ml$^{-1}$ were able to inhibit antigen binding and 'bridging' between the antibodies (experiments 1 and 2, Table VII). However, when C595 antibody was the 'capture' antibody, the peptide P(1–20), but not smaller peptides, produced 97 and 96% inhibition of antigen binding at 500 µg ml$^{-1}$ (experiments 1 and 2), and even at 5 µg ml$^{-1}$, P(1–20) produced 60% inhibition of antigen binding (experiment 3, Table VII). These findings suggest that the epitope for the antibody, C595, resides in the protein core of epithelial mucins.

Finally, it should be noted that an IgM antibody such as NCRC-11, on binding to a macromolecular antigen with repeating epitopes, may achieve a multiple binding bonus which renders competition by a peptide much less effective than with an IgG antibody like C595. Thus, it cannot be concluded from the experiments in Table VII that the NCRC-11 antibody defines a non-protein epitope.

**Table VII** Inhibition of binding of epithelial mucin to antibody by pre-incubation of antibody with synthetic peptides

| *Expt* | *'Capture' antibody* | *Conc. of inhibiting peptide (µg ml$^{-1}$)* | *Percentage inhibition (mean±s.d.) of binding of epithelial mucin by pre-incubation of 'capture' antibody with peptide* PBS | P(12–20) | P(7–20) | P(1–20) |
|---|---|---|---|---|---|---|
| 1 | C595 | 500 | 0±4 | | −40±12 | 97±2 |
| | NCRC-11 | 500 | 0±5 | | −22±12 | −12±7 |
| 2 | C595 | 500 | 0±4 | | −11±3 | 96±2 |
| | NCRC-11 | 500 | 0±11 | | −32±6 | −12±7 |
| 3 | C595 | 50 | 0±6 | 12±2 | 8±8 | 83±5 |
| | | 15 | 0±5 | 11±5 | 3±6 | 78±3 |
| | | 5 | 0±2 | 4±11 | 5±5 | 60±3 |

### Discussion

Production of a 'second generation' monoclonal antibody against a urinary epithelial mucin antigen which has been immuno-affinity purified using an antibody of the 'first generation', has yielded an antibody, C595, of virtually identical specificity to its 'parent' but of the immunoglobulin IgG class rather than being an IgM antibody. In fact, the data in Tables I to V and Figures 1 and 2 all serve to emphasise the similarities between the two antibodies, C595 and NCRC-11. The preliminary survey of antibody reactivity with normal tissues and breast tumours, by immunohistology (Table I) or in subcellular membrane binding assays (Table II), revealed little difference between C595 and NCRC-11 antibodies. Furthermore, in immunoblotting experiments, both antibodies bound to high molecular weight antigens (Figure 1) which were of high bouyant density (Figure 2). The reactivity of the two antibodies in various immunoassays also exemplified their siimilarities (Tables III to V).

In Table VII, the binding of urinary mucin to C595 antibody was clearly inhibited by incubation of antibody with the synthetic peptide, P(1–20), which represents the complete peptide motif which is repeated in epithelial mucins (Gendler *et al.*, 1988). This would indicate that the epitope for C595 is expressed within the protein core of the mucin. Since C595 failed react with all but the largest peptide, then it might be anticipated that its epitope will be found within the first half of the peptide P(1–20), within the sequence P D T R P A P G S T (Table VI).

It appeared not to be possible to modify antigen binding to the antibody NCRC-11 by equivalent incubation with the synthetic peptides (Table VII). This might be taken to suggest that NCRC-11 antibody reacts with a non-protein determinant perhaps expressed within the carbohydrate domains of the mucin, rather than in the protein core. Alternatively, synthetic peptides may be less potent inhibitors of multivalent IgM antibodies (e.g. NCRC-11) as compared with IgG antibodies such as C595. Most recent results have demonstrated that both C595 and NCRC-11 antibodies bind to peptides which have been synthesised on a solid phase ('tethered' peptides), so that the tests described in Table VII may have been inappropriately designed to reveal the interaction of peptide determinants with NCRC-11 antibodies of the IgM class. Studies are in progress to localise the epitopes for C595 and NCRC-11 antibodies more precisely within the protein core sequence.

Since the protein core of epithelial mucins consists of

**Table VI** Epithelial mucin core – Antibody reactivity with synthetic peptides

| *Peptide* | *Amino acid number* 1 5 10 15 20 | *Reaction of antibody* NCRC-11 | C595 |
|---|---|---|---|
| P (12–20) | P P A H G V T S A | – | – |
| P (7–20) | P G S T A P P A H G V T S A | – | – |
| P (1–20) | P D T R P A P G S T A P P A H G V T S A | – | + |
| | * ** ** | | |

*Potential glycosylation sites.

tandem repeats of 20 amino acid peptide, this provides an adequate model incorporating the multiple repeats of the C595 and NCRC-11 defined epitopes which are required for the isolated antigen to 'bridge' homologous and heterologous combinations of these two antibodies in IRMAs (Table IV). Comparably, the close proximity of the peptide regions which are likely to express the epitopes for C595 and NCRC-11 antibodies would explain why C595 antibody was almost as effective as NCRC-11 antibody at inhibiting radiolabelled NCRC-11 antibody binding to antigen (Table V).

Evidence is now accumulating that a number of antibodies produced in different laboratories react with the protein core of epithelial mucins rather than with the carbohydrate side chains (e.g. antibodies HMFG-1, HMFG-2, SM-3; Burchell *et al.*, 1987). Preferential reactivity of anti-core antibodies for tumours may be achieved if the peptide core is more accessible in malignancy-derived mucins. This is feasible since in tumours, aberrant or incomplete glycosylation as well as the action of tumour-associated glycosidases and glycosyltransferases, may well generate core epitopes which are more cryptically expressed (i.e. less accessible) in normal tissue mucins. Staining of tumour tissue sections by NCRC-11 and C595 antibodies certainly displays wide heterogeneity both within and between specimens, and this may reflect differences in the accessibility or exposure of mucin protein core epitiopes in malignant tissues. Also, staining when observed in normal tissues (and only then, confined to the luminal surface of specialised epithelia) does not appear to attain the same intensity as can be found throughout some, but not necessarily all, tumour tissue sections – thus, the total antigen content or load in breast carcinoma tissue can be considerably greater than in the corresponding normal tissue. If irregular staining of tumour cells is due to incomplete or defective synthesis or carbohydrate chains, then it follows that there may also be epitopes generated in tumour mucin oligosaccharides which are preferentially associated with tumours. The fact remains, however, that with the insight gained upon the nature of the protein core and its antibody defined epitopes, it becomes a feasible objective to design strategies for the production of new antibodies with increased tumour reactivity using more rational approaches than was formerly possible.

These studies were supported by the Cancer Research Campaign. M. Sekowski is thanked for preparing the purified NCRC-11 and C595 antibodies. Helpful discussions with Dr Joy Burchell (Imperial Cancer Research Fund Laboratories), concerning antibody reactivity with synthetic peptides, are gratefully acknowledged.

### References

BURCHELL, J., DUBIN, H. & TAYLOR-PAPADIMITRIOU, J. (1983). Complexity of expression of antigenic determinants recognised by monoclonal antibodies HMFG-1 and HMFG-2, in normal and malignant human mammary epithelial cells. *J. Immunol.*, **131**, 508.

BURCHELL, J., GENDLER, S., TAYLOR-PAPADIMITRIOU, J. & 4 others (1987). Development and characterization of breast cancer reactive monoclonal antibodies directed to the core protein of the human milk mucin. *Cancer Res.*, **47**, 5476.

ELLIS, I.O., ROBINS, R.A., ELSTON, C.W., BLAMEY, R.W., FERRY, B. & BALDWIN, R.W. (1984). A monoclonal antibody, NCRC-11, raised to human breast carcinoma. I. Production and immunohistological characterization. *Histopathology*, **8**, 501.

GENDLER, S., TAYLOR-PAPADIMITRIOU, J., DUHIG, T., ROTHBARD, J. & BURCHELL, J. (1988). A highly immunogenic region of a human polymorphic epithelial mucin expressed by carcinomas is made up of tandem repeats. *J. Biol. Chem.*, **263**, 12820.

HILKENS, J., BUIJS, F., HILGERS, J. & 4 others (1984). Monoclonal antibodies against human milk-fat globule membranes detecting differentiation antigens of the mammary gland and its tumors. *Int. J. Cancer*, **34**, 197.

JENSENIUS, J.C. & WILLIAMS, A.F. (1974). The binding of anti-immunoglobulin antibodies to rat thymocytes and thoracic duct lymphocytes. *Eur. J. Immunol.*, **4**, 91.

KRUPEY, J., WILSON, T., FREEDMAN, S.O. & GOLD, P. (1972). The preparation of purified carcinoembryonic antigen of the human digestive system from large quantities of tumour tissue. *Immunochemistry*, **9**, 617.

KUFE, D., HAYES, D. & ABE, M. (1988). Monoclonal antibody assays for breast cancer. In *Cancer Diagnosis in vitro Using Monoclonal Antibodies*, Immunology Series Volume 39, Kupchik, H.Z. (ed.) p. 67. M. Dekker: New York.

LAEMMLI, U.K. (1970). Cleavage of structural proteins during the assembly of the head of bacteriophage T4. *Nature*, **227**, 680.

LAN, M.S., BAST, R.C., COLNAGHI, M.I. & 4 others (1987). Co-expression of human cancer-associated epitopes on mucin molecules. *Int. J. Cancer*, **39**, 68.

PRICE, M.R. (1988). High molecular weight epithelial mucins as markers in breast cancer. *Eur. J. Cancer Clin. Oncol.*, **24**, 1799.

PRICE, M.R., CROCKER, G., EDWARDS, S. & 6 others (1987*a*). Identification of a monoclonal antibody defined breast carcinoma antigen in body fluids. *Eur. J. Cancer Clin. Oncol.*, **23**, 1169.

PRICE, M.R., EDWARDS, S., OWAINATI, A. & 4 others (1985). Multiple epitopes on a human breast carcinoma associated antigen. *Int. J. Cancer*. **36**, 567.

PRICE, M.R., EDWARDS, S. & BALDWIN, R.W. (1986*a*). Association of the Y hapten with glycoproteins, glycolipids and carcinoembryonic antigen in colorectal carcinoma. *Cancer Lett.*, **33**, 83.

PRICE, M.R., EDWARDS, S., JACOBS, E., PAWLUCZYK, I.Z.A., BYERS, V.S. & BALDWIN, R.W. (1987*b*). Mapping of monoclonal antibody defined epitopes associated with carcinoembryonic antigen. *Cancer Immunol. Immunother.*, **25**, 10.

PRICE, M.R., EDWARDS, S., POWELL, M. & BALDWIN, R.W. (1986*b*). Epitope analysis of monoclonal antibody NCRC-11 defined antigen isolated from human ovarian and breast carcinoma. *Br. J. Cancer*, **54**, 393.

SEKINE, H., OHNO, T. & KUFE, D.W. (1985). Purification and characterization of a high molecular weight glycoprotein detectable in human milk and breast carcinoma. *J. Immunol.*, **135**, 3610.

TOWBIN, H., STAEHLIN, T. & GORDON, J. (1979). Electrophoretic transfer of proteins from polyacrylamide gels to nitrocellulose sheets: procedure and some applications. *Proc. Natl Acad. Sci. USA*, **76**, 4350.